# On the Interpretation and Use of Mediation: Multiple Perspectives on Mediation Analysis

**DOI:** 10.3389/fpsyg.2017.01984

**Published:** 2017-11-15

**Authors:** Robert Agler, Paul De Boeck

**Affiliations:** ^1^Department of Psychology, Ohio State University, Columbus, OH, United States; ^2^Division of Epidemiology, College of Public Health, Ohio State University, Columbus, OH, United States; ^3^Department of Psychology, KU Leuven, Leuven, Belgium

**Keywords:** mediation, causation, total effect, direct effect, indirect effect

## Abstract

Mediation analysis has become a very popular approach in psychology, and it is one that is associated with multiple perspectives that are often at odds, often implicitly. Explicitly discussing these perspectives and their motivations, advantages, and disadvantages can help to provide clarity to conversations and research regarding the use and refinement of mediation models. We discuss five such pairs of perspectives on mediation analysis, their associated advantages and disadvantages, and their implications: with vs. without a mediation hypothesis, specific effects vs. a global model, directness vs. indirectness of causation, effect size vs. null hypothesis testing, and hypothesized vs. alternative explanations. Discussion of the perspectives is facilitated by a small simulation study. Some philosophical and linguistic considerations are briefly discussed, as well as some other perspectives we do not develop here.

## Introduction

Without respect to a given statistical model, mediation processes are framed in terms of intermediate variables between an independent variable and a dependent variable, with a minimum of three variables required in total: *X*, *M*, and *Y*, where *X* is the independent variable (IV), *Y* is the dependent variable (DV), and *M* is the (hypothesized) mediator variable that is supposed to transmit the causal effect of *X* to *Y*. The total effect of *X* on *Y* is referred to as the total effect (*TE*), and that effect is then partitioned into a combination of a direct effect (DE) of *X on Y*, and an indirect effect (*IE*) of *X* on *Y* that is transmitted through *M*. In other words, the relationship between *X* and *Y* is decomposed into a direct link and an indirect link.

While the conceptual model of mediation is straight-forward, applying it is much less so (Bullock et al., 2010). There are multiple schools of thought and discussions regarding mediation that provide detailed arguments and criteria regarding mediation claims for specific models or sets of assumptions (e.g., Baron and Kenny, 1986; Kraemer et al., 2002; Jo, 2008; Pearl, 2009; Imai et al., 2010). As still further evidence of the difficulty of making mediation claims, parameter bias, and sensitivity have emerged as common concerns (e.g., Sobel, 2008; Imai et al., 2010; VanderWeele, 2010; Fritz et al., 2016), as has statistical power for testing both indirect (e.g., Shrout and Bolger, 2002; Fritz and MacKinnon, 2007; Preacher and Hayes, 2008) and total effects (Kenny and Judd, 2014; Loeys et al., 2015; O'Rourke and MacKinnon, 2015).

Relatively untouched is that there are cross-cutting concerns related to the fact that what is considered appropriate for a mediation claim depends not only on statistical and theoretical criteria, but also on the experience, assumptions, needs, and general point of view of a researcher. Some perspectives may be more often correct than others (e.g., more tenable assumptions, better clarification of what constitutes a mediator, etc.), but all perspectives and models used by researchers are necessarily incomplete and unable to fully capture all considerations necessary for conducting research, leaving some approaches ill-suited for certain tasks. This is in line with a recent article by Gelman and Hennig (2017), who note that while the tendency in the literature is to find and formulate one best approach based on seemingly objective criteria there is nonetheless unavoidable subjectivity involved in any statistical decision. Researchers always view only a subset of reality, and rather than denying this it is advantageous—even necessary—to embrace that there are multiple perspectives relevant to any statistical discussion.

The aim of the article is not to propose new approaches or to criticize existing approaches, but to explain that the existence and use of multiple perspectives is both useful and sensible for mediation analysis. We use the term *mediation* in the general sense that a mediation model *explains values of Y* as indirectly caused by values of *X*, without favoring any specific statistical model or set of identifying assumptions. The three variables may be exhaustive, or a subset of much larger set of variables. As we discuss, there can be value in different and divergent considerations and convergence is not required or uniformly advantageous. Our points here are more general than any specific statistical model (and their *IE, DE*, and *TE* estimates and tests), but there are a few points that require we first review simple mediation models as estimated by ordinary least squares linear regression. We will then take the concept of mediation to an extreme with a time-series example, using the example to illustrate and discuss the various perspectives, not as a representative case but to clarify some issues.

## Mediation with linear regression

**Figure 1:**
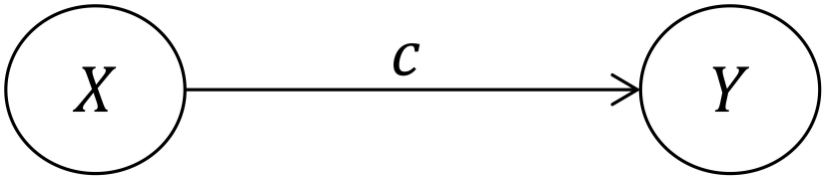
Effect of *X* and *Y* without considering mediation.

**Figure 2:**
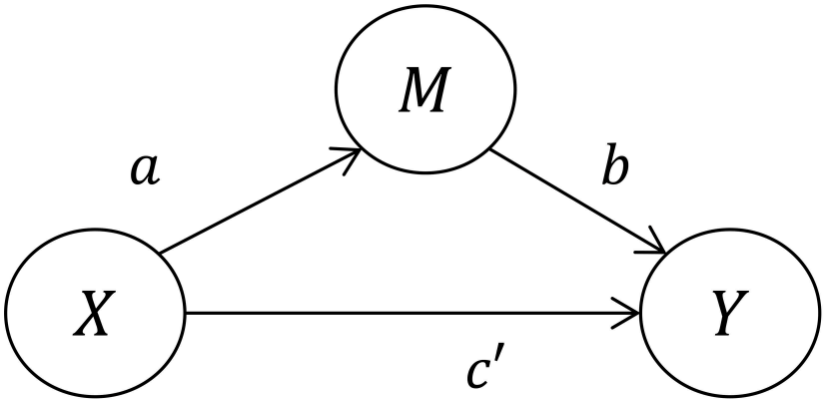
Effect of *X* on *Y* including mediation.

Within a regression framework, the population parameters *a, b, c*, and *c*′ (Figures 1, 2) are estimated not with a single statistical model, but rather a set of either two or three individual regression models. We say two or three because the first, Model 1, is somewhat controversial and is not always necessary (Kenny and Judd, 2014). This model yields the sample regression weight *c* as an estimate of the *TE:*

(1) $$Y=i_{1}+cX+e_{1}$$

Models 2 and 3 are used to estimate the *DE* and *IE*. Specifically, the *DE* is presented as the path from *X* to *Y*, *c*′. The *IE* is estimated by the product of the path from *X* to *M* (Model 2) and the path from *M* to *Y* (Model 3), i.e., the product of the regression weights *a* and *b*. The equations for these two models are as follows:

(2) $$M=i_{2}+aX+e_{2}$$

(3) $$Y=i_{3}+c\prime X+bM+e_{3}$$

Together, these two models yield the direct effect, *c*′, as well as the indirect effect *ab*. Further, the summation of these two effects is equal to the total effect, i.e., *c* = *c*′+*ab*. Assuming no missing data and a saturated model (as in the case of Equations 2 and 3) this value of *c* is equal to that provided by Model 1.

The total effect can then be inferred in two different ways, either based on Figure 1 (Model 1) or on Figure 2 (a combination of Models 2 and 3), but as we will discuss there are important conceptual differences between these two numerically identical total effects. We will refer to the *TE* associated with Figure 1 as *TE*_*1*_, and the *TE* associated with Figure 2 as *TE*_*2*_.

## A time series example

To take the concept of mediation to an extreme, imagine a stationary autoregressive process for *T* equidistant time points (e.g., *T* consecutive days) with a lag of 1 as in the most simple autoregressive time series model, i.e., *AR*(1). In such a model the expected correlation between consecutive observations is stable (stationary), and the model is equivalent with a full and exclusively serial mediation model without any direct effect. *X* is measured at *t* = 1 and *Y* is a measured at *t* = *T*. The independent variable *X* has an effect on *M*_*t*__= 2_, which in turn has an effect on *M*_*t*__= 3_, and so on up to *M*_*t*__= T−1_ having an effect on *Y* at *t* = *T*. In mediation terms, there are *T*-2 mediators, from *M*_*t*__= 2_ to *M*_*T*__−1_, with an effect only on the next mediator and finally on *Y*. Although this kind of mediation is an extreme case compared with the typical simple mediation model, it is nonetheless mediation in the sense that all effects are transmitted by way of an intervening effect. As a result, regardless of the time scale, the *TE* always equals the *IE*. Although extreme, such a model is a reasonable one for some time series data, e.g., it seems quite realistic that one's general mood (as distinct from ephemeral emotional states) of today mediates between one's mood of yesterday and one's mood of tomorrow. For some variables, there may be also an effect from earlier values than the previous measurement, i.e., longer lags, but such a more complex process is still a mediation process.

To help make our points more concrete we conducted a small-scale simulation. We generated data for 3, 10, 50, or 100 time points with a constant correlation of 0.10, 0.50, or 0.90 between consecutive time points, and with *N* = 10, 50, or 100 for each, for a total of 36 conditions. Initial time points were drawn from a standard normal distribution. We generated 500 replications per condition. All tests were done using 5,000 bootstraps and α = 0.05. These results are shown in Table 1. One can easily see that rejections of the null hypothesis for the total effect *TE*_*1*_ scarcely exceed the α level in nearly all conditions, which is unsurprising because of the near zero magnitude of the total effect. The only exceptions to these low rejections rates were for *N* = 10—but this is due to bootstrapping underestimating the standard error here for such small sample sizes—and for cases where the *TE* was of appreciable magnitude, i.e., for *T* = 3 and *r* = 0.5 or 0.9 or *T* = 10 and *r* = 0.9 (*TE* = *IE* = 0.25, 0.81, and 0.38742, respectively). For such large effects the *TE*_*1*_ is easily rejected. In contrast, the indirect effect is almost always significant, and the rejection rates are always greater than those of the *TE*_*1*_, even when the true size of the indirect effect is extremely small (as small as the true total effect). For nearly all cases where *r* = 0.5 or 0.9 the test of the *IE* exhibited higher power than the test of the *TE*_*1*_, with the minor caveat that for *r* = 0.5 and *N* = 10 the difference was minimal. In total, for 20 conditions of the 36 we considered here, rejection rates were 89–100%, with the observed power advantage for the *IE* relative to the *TE*_*1*_ as great as 94% higher (6 vs. 100%) when the *TE*_*1*_ is small, e.g., when *T* = 50 or 100. We will use this illustration to elaborate on the different perspectives on mediation, and specific aspects of the results will be focused on as necessary for the perspectives we discuss.

**Table 1:** Simulation results.

**T**	***r***	***TE* = *IE***	**N**	***TE*% *p* < 0.05**	***IE*% *p* < 0.05**
*3*	*0.1*	*0.01*	10	6.4	0.4
			50	5.8	1.2
			100	5.8	4.6
	0.5	0.25	10	15.6	14.0
			50	43.6	91.8
			100	72.0	99.8
	0.9	0.81	10	89.6	95.0
			50	100	100
			100	100	100
10	0.1	1e-09	10	06.8	0
			50	7.4	0.8
			100	05.2	2.2
	0.5	0.00195	10	10.4	12.0
			50	6.4	88.8
			100	06.6	100
	0.9	0.38742	10	25.2	92.2
			50	80.2	100
			100	98.8	100
50	0.1	1e-49	10	13.4	0.8
			50	5.6	1.2
			100	5.4	1.6
	0.5	1.78e-15	10	6.8	10.0
			50	7.6	93.8
			100	5.0	100
	0.9	0.00573	10	10.0	94.8
			50	6.4	100
			100	5.8	100
100	0.1	1e-99	10	10.4	1.0
			50	6.0	1.6
			100	4.2	3.8
	0.5	1.58e-30	10	9.4	16.6
			50	5.6	92.6
			100	6.0	100
	0.9	0.00003	10	10.2	94.6
			50	6.6	100
			100	6.4	100

*Results of a small-scale simulation conducted to illustrate perspectives regarding mediation, with 500 replications per condition*.

## Five pairs of perspectives

Each of the five pairs of perspectives we discuss here offers a choice regarding how to view, use, and study mediation models. Each of the perspectives we discuss here has its own merits, and we do not mean to imply that any perspective or approach we discuss here is “better”—there are simply too many criteria to exhaust to evaluate such a claim, and researchers must work within the context of the problem at hand to decide what is most appropriate.

We dichotomize and treat each perspective both within and between pairs as largely independent for the purposes of explication, but there are many points of intersection and we do not wish to imply an absence of a middle ground or that each perspective from a given pair cannot be meaningfully integrated. The perspectives we discuss here are not meant to be exhaustive, and were selected because of their relevance to common topics in the mediation literature. No pair of perspectives is strictly limited to any one topic, as the various discussions regarding mediation are each better understood when looked at from multiple angles. A brief summary of each pair of perspectives we discuss is provided in Table 2, as well as a few example areas of research where the perspectives are relevant.

**Table 2:** Comparison of perspectives.

	**Summary**	**Rationale**	**Notes**	**Relevant to discussions and debates about**
With vs. without a mediation hypothesis				*TE* power False-positive psychology
	Statistical significance of the *TE* is irrelevant for testing mediation	A mediation hypothesis is required before testing for indirect effects The test of *IE* often has greater power than the test of the *TE_*1*_*, and requiring *TE_*1*_* be significant ignores this, as well as the risk of opposing direct and indirect	The basis of both NHST and theory-driven research necessitate that tests are done carefully, and statistical significance is not “fished” for	
	The *TE* should be significant before testing mediation	The basis of both NHST and theory-driven research necessitate that tests are done carefully, and statistical significance is not “fished” for by moving to a *IE* test after non-significance of *TE_*1*_* test	Does not apply to exploratory research conducted in a manner that is in keeping with best practices	
Specific Effects vs. Global Model				SEM vs. regression Network models
	Effects perspective focuses on individual parameters, with inferences based on their presence and/or strength	Often it is only necessary to show that an effect exists, generally in support of a given theoretical framework	Model-constraints that may bias parameters are avoided, but runs the risk of overfitting and poor replication rates due to larger standard errors	
	Statistical models are the focus, with mediation effect(s) a subset of the global set of relationships	Effects do not occur in isolation, and are instead a part of a larger set of causes, effects, and boundary conditions	Higher precision and better replication if model assumptions hold, but biased parameter estimates if they do not. Interpretation of the effects is also conditional on the global model used	
Significance testing vs. Effect sizes				Theory building vs. practical application
	Null hypothesis significance testing (NHST) is used as the criteria to evaluate an effect	Priority is to distinguish an effect from noise	Ignores gradation of effects and does not address proportion of outcome variance explained	
	Effect sizes and confidence are used to evaluate an effect	Establishing presence is insufficient because uncertainty and magnitude affect both replication and relevance of a given effect	Meaningful effects may be ignored because they seem too small to matter in practice, but are nonetheless important when aggregated across many people or for theory building	
Direct vs. Indirect Effects				Appropriateness of indirect effects
	A directness perspective focuses establishing direct causes	For some problems and situations one may want to know direct causes, intermediate steps leave room for interference	Has the advantage that one does not need to count on uncertain intermediate steps, but potentially ignores suppression effects due to other variables	
	An indirectness perspective focuses on understanding the intermediate steps between IV and DV, between a cause and its effect	For almost any given cause and effect encountered there are intermediate effects, and explaining and understanding these effects provides more information about the original cause and effect mechanisms which may themselves be more relevant to the outcome	Provides a fuller picture of the relationship between X and Y, but at much greater risk of making incorrect inferences and explanations	
Hypothesized vs. Alternative explanations				False-positive psychology Parameter sensitivity
	Focus is on confirmatory evidence and supporting a mediation hypothesis	Past research should guide future research, increasing speed and ideally efficiency of research	Restricted explanation search that runs the risk of attempting to support previous findings and neglecting more accurate alternative explanations	
	Focus is on testing alternative hypotheses that conflict with a specific mediation hypothesis	Evidence in support of a mediation claim is stronger if it can be shown that plausible alternative explanations do not hold	Infinite number of alternative explanations, both reasonable and unreasonable, and often no clear stopping rule for what constitutes an adequate search	

## With vs. without a mediation hypothesis

A common concern that has emerged in the mediation literature is whether or not *TE*_*1*_ should be required before testing indirect effects. Given that the reason researchers use mediation analysis is to test for indirect effects, whether or not there is a total effect can seem an irrelevant preliminary condition. Our time-series example is one example of why the presence of *TE*_*1*_ is not required for an indirect effect to be detected with a null hypothesis test, but even in more mundane cases involving three variables the *IE* test has greater power than the *TE*_*1*_ test under some parameter configurations (Rucker et al., 2011; Kenny and Judd, 2014; Loeys et al., 2015; O'Rourke and MacKinnon, 2015). Further, two competing effects can suppress each other (MacKinnon et al., 2000) such that two roughly equal (and potentially large) direct and indirect effects of opposing direction can result in a near-zero total effect. As can be seen in Table 1, a large proportion of the tests of the *IE* were significant even when the corresponding test of the *TE*_*1*_ was not significant. These are not new findings, but they illustrate that even for extremely small effect sizes such as, at the bottom of Table 1 (e.g., 1.58e-30) the *IE* is significant. Given a mediation hypothesis there is then no need to consider the significance of the *TE*_*1*_ because it is irrelevant to the presence of an *IE*, as the *IE* is estimated by different statistical models than *TE*_*1*_ is and a mediation hypothesis refers solely to the *IE* (though a more general causal relationship may be hypothesized to include both).

However, such work should not be taken as a blanket justification for testing the *IE* in the absence of *TE*_*1*_ if there is not an *a priori* hypothesized indirect effect. While there is great value and need for exploratory research (with later replication and validation in a separate study) and we do not wish to discourage such practices, if the *XY* relationship is not significant based on Model 1 then one is likely better served by staying with the null hypothesis of no relationship because of the increased risk of false positives associated with so-called “fishing expeditions” (Wagenmakers et al., 2011). Although a non-significant relationship does not exclude the possibility that there is a true and perhaps mediated relationship between *X* and *Y*—the world is full of relationships that cannot be differentiated from noise without consideration of indirect effects—a preference for parsimony and a desire to avoid false positives would suggest that one does not generate additional explanations for relationships that are not significant when first tested. Although the results shown in Table 1 show that a large proportion of indirect are significant in the absence of a significant *TE*_*1*_ it would not be a good idea to follow up all non-significant correlations, regression weights, *F*-tests, *t*-tests, etc. with a *post-hoc* mediation analysis and then attempting to explain it after the results are known (Kerr, 1998). When working with real data there are simply too many alternative explanations to consider. Absent an *a priori* hypothesis, the Judd and Kenny (1981) and Baron and Kenny (1986) condition requiring that the relationship between *X* and *Y* be significant makes sense.

The two perspectives represent two different and contrasting lines of reasoning and motivations—either the study is based on a mediation hypothesis or it is not. If it is, there is no preliminary condition regarding the total effect because it is irrelevant to whether or not an indirect effect may be present. It is simply necessary to conduct the appropriate test for the indirect effect. If however there was no pre-specified hypothesis, the logic of null hypothesis significance testing (NHST) requires that one stays with the conclusion of no relationship if the null hypothesis is not rejected by the data rather than conducting additional unplanned tests (with the caveat that appropriate corrections for multiple comparisons may be employed).

## Specific effects vs. global model

To put it colloquially, this pair of perspectives refers to whether one is interested in the forest or in the tree when investigating mediation. An effect-focused approach implies that a global model for all relationships is less important, and that one focuses instead on the tests of the effects of interest. These effects can be tested within a global statistical model (i.e., one can be interested in specific effects while still estimating all relationships), or from separate regression models. In the latter case, the global model is then primarily a conceptual one because there is not one statistical model to be used for estimation of the effects. For example, when using separate regressions the indirect effect is the product of two parameters from different statistical models, and while *TE*_*1*_ is an effect in one model, *TE*_*2*_ is a composite of two effects that stem from two separate models.

In contrast, a globally focused approach implies formulating and testing a global model for all variables, evaluating it based on relevant criteria (e.g., model fit, theoretical defensibility). The various examples of network models are examples of global models (Salter-Townshend et al., 2012), but most commonly in the social sciences global models are realized using a structural equation model approach (SEM) for the covariance of the three variables, with or without making use of any latent variables (Iacobucci et al., 2007; MacKinnon, 2008). If latent variables are used then there is the advantage of correcting for measurement error, but it is not necessary to use latent variables in a global model. Within the model, the specific mediation effect can be derived as a product of single path effects (e.g., Rijnhart et al., 2017).

The choice between, and discussions regarding, these two approaches comes with a few relevant considerations. First, there is the matter of model saturation (i.e., the same number of estimated parameters as there are variables). For the simple situation of one mediator variable and thus three variables in total, and effects described by *a, b*, and *c*′, the global model is a saturated model, and as a result the point estimate of the indirect effect is the same whether one uses different regression models or one global SEM. To some degree then the matter of specific effects vs. the global model distinction is irrelevant because simple mediation models are saturated. However, when the mediation relationships are more complex the global model is no longer necessarily a saturated model. For example, a two-mediator model is either a serial or parallel mediator model, with the former having a path between the two mediators and the latter not (Hayes, 2013). As such, a parallel two-mediator model is not saturated whereas a serial two-mediator model is. In general, from a global model perspective one would first want to test the goodness of fit of the global model, before a particular mediation effect is considered at all because the effects are conditional on the model.

Second, the power anomaly discussed in recent work reflects an effect-focused perspective based on separate regressions and vanishes when one focuses on the effect within a global statistical model, where the covariance between *X* and *Y* is simply a descriptive statistic used for model estimation and not a parameter (i.e., not a total effect to estimate). The total effect is estimated through two within-model effects. *TE*_*1*_ is one observed covariance among the other observed covariance measures to be explained with the model. Further, instead of two separate *TE* estimates (stemming from separate regressions), there is only one *TE* to be considered: *TE*_2_ as estimated from the model *TE*_*model*_:

(4) $$TE_{SEM}=a^{*}\times b^{*}+c\prime^{*}$$

Where a^*^, b^*^, and *c*′^*^ are model parameters. Of course, when *c*′^*^ = 0, then $TE_{SEM}=a^{*}\times b^{*}$.

Although the point estimates of *TE*_*1*_ and *TE*_*2*_ are equal for a simple mediation model, neither their associated models nor their sampling distributions are. For example, it is well known that the sampling distribution of the indirect effect estimate is skewed unless the sample size is extremely large (MacKinnon et al., 2004) and this also applies when estimated from a global model (the product of a^*^ and b^*^). The skewness is inherent to the distribution of a product, and this transfers to the distribution of *TE*_*2*_ whether estimated based on a global model or through separate regressions. In contrast, there is no reason to expect skewness in the sampling distribution of *TE*_*1*_ because it is a simple parameter in Equation (2) and Figure 1, and not a product of two parameters.

The study of mediation is almost entirely effect-focused because the substantive hypotheses are mostly about particular mediation effects and their presence or not (typically defined by statistical significance), and so a global model test makes less sense from that perspective. This is particularly true because perfect model fit for the covariance of the variables is guaranteed in a simple mediation model with just the three variables *X, M*, and *Y*, despite a simple mediation model being almost certainly incomplete (Baron and Kenny, 1986; Sobel, 2008). If one is primarily interested in the effects, it further makes sense to be liberal on the model side because model constraints can lead to bias in the parameter estimates (e.g., forcing a genuine *DE* to be equal to 0 will bias the *IE* estimate) and the standard errors.

In contrast, one can expect a model testing approach to prevail in a global process theory that describes the set of variable relationships as a whole. In such a case an SEM makes more sense, and within the model one or more indirect effects are tested (e.g., van Harmelen et al., 2016). The time series example is another case where a global model approach makes sense. From an effects perspective the mediation effect for a series of 100 would be a product of 99 parameters and the direct effect would span 99 time intervals, but these would be of relatively little interest or importance. Instead it is the model that matters, and within the model the autoregressive parameter is of interest (and not the *IE* as a product of all these autoregressive parameters as we did for the simulation study). In a simple autoregressive model with lag 1, i.e., *AR*(*1*), *a* = *b* (and so on, depending on the number of time points), and *c*′ = 0. The *AR(1)* autoregressive model characterizes the relevant system, e.g., mood, self-esteem, etc.

As before, the two perspectives are both meaningful. One can either be interested in a global model for the relationships or one can give priority to the effects and minimize the importance of the overall model. The fewer modeling assumptions associated with an effects-perspective may lead to poorer precision and replication (e.g., larger standard errors and greater risk of overfitting), but model-based constraints are avoided. Conversely, making more assumptions leads to better precision and possibly to better replication (if the model constraints are valid). One can also make the statistical model more in line with the theoretical model in order to impose a stronger test of a theory. However, the assumptions are made at the risk of distorted parameter estimates, and the effect estimates are also conditional on the global model they belong to, which can complicate interpretation somewhat. Therefore, it can make sense to stay with separate regression analyses without a test of the global model.

## Effect size vs. null hypothesis testing

Based on criticism of NHST (e.g., Kline, 2004), effect size and confidence intervals have been proposed as an alternative approach to statistical analyses (e.g., Cumming, 2012). These points have emerged in the mediation literature as well, with mediation-specific effect sizes discussed and proposed (e.g., Kraemer et al., 2008; Preacher and Kelley, 2011), and bootstrapped confidence intervals are now the standard for testing indirect effects (e.g., Shrout and Bolger, 2002; Hayes, 2013; Hayes and Scharkow, 2013).

Numerous effect size indices have been proposed for the *IE*, and these indices may take the form of either variance in the DV explained or in terms of the relative effects as in the case of the ratio *ab/c*′ (an excellent review may be found in Preacher and Kelley, 2011; note however the specific effect size proposed by these authors was later shown to be based on incorrect calculations; Wen and Fan, 2015). As it is not our intention to promote one particular measure, but rather to make a general point regarding effect size vs. null hypothesis testing perspectives, we simply use the product of the standardized *a* and *b* coefficients.

In the largest time series model illustrated previously, the indirect effect is a product of 99 terms, and as a result the expected effect size with an autoregressive coefficient of 0.90 is still a negligible 0.00003. Even so, this extremely small effect can easily lead to a rejection of the null hypothesis when the *IE* is tested, as illustrated in Table 1. The confidence intervals are very narrow for such a small effect, but they do not include zero. In practice, such an example would represent mediation from the NHST perspective (supported by the confidence intervals) and it could potentially be a very meaningful finding, but from the effect size perspective the effect may seem too small to be accepted or worth consideration for any practical decisions. Both points of view make sense. There is clearly mediation in the time series example, but the resulting effect is negligible in terms of the variance explained at time 100. The distance between *X* and *Y* is too large for a difference in *X* to make a difference for *Y* while in fact the underlying process is clearly a mediation process with possibly a very large magnitude from time point to time point (i.e., as small as 0.9).

As before, neither perspective is strictly superior because both perspectives have advantages and disadvantages. One possible problem when approaching mediation from the NHST perspective is that it is perhaps too attractive to look for possible mediators between *X* and *Y* after failing to reject the initial null hypothesis because of the work showing that a test of the *IE* has higher power, in particular given the high rates at which the *TE* is not rejected but the *IE* is as shown in Table 1 (to be clear, a strict NHST perspective would not permit such an approach, as discussed previously). Other problems are the dichotomous view on mediation (mediation vs. no mediation) while effects are in fact graded (Cumming, 2012), and the fact that rejection of the null hypothesis does not speak to how well the variance of *Y* is explained.

The effect size logic has its own drawbacks as well, of course. Competing indirect effects, regardless of size, can cancel each other out (note this holds true for all effects in a mediation model, e.g., *a* may be small because of competing effects from *X* to *M*). Another issue is that the effect size is commonly expressed in a relative way (e.g., in terms of the standard deviation of the DV or a percentage explained variance) and therefore it depends on the variance in the sample and on other factors in the study that raise questions about the appropriateness of many mediation effect sizes (Preacher and Kelley, 2011). What constitutes a relevant effect size is also not always immediately clear, as it depends immensely on the problem at hand, e.g., what the dependent variable is, how easily manipulated the independent variable(s) are, etc. A further complicating factor is that most psychological variables have arbitrary units, such as, units on a point-scale or response option numerical anchors for a questionnaire. For variables with natural units, such as, the number of deadly accidents on the road or years of life after a medical intervention, one would not need a standard deviation or a percentage of variance to express the effect size in a meaningful way.

As with the previous perspectives, these two perspectives throw light on two relevant but different aspects of the same underlying reality. The null hypothesis test is a test of a hypothesized process and whether it can be differentiated from noise, whereas the effect size and confidence intervals tell us how large the result of the process is and what the width of the uncertainty is. Not all processes have results of a substantial size—and this is clear in the time-series example we showed previously—but even an extremely small effect can be meaningful as the indication of a process.

## Directness vs. indirectness

Another pair of perspectives depends upon the semantics of causality. In both linguistics (e.g., Shibatani, 2001) and in law (e.g., Hart and Honore, 1985), directness is an enhancer of causal interpretation, and a remote cause is considered less of a cause or even no cause at all. In contrast, in the psychological literature a causal interpretation is supported when there is evidence for an intermediate psychological or biological process and thus for some indirectness. Causality claims seem supported if one can specify through which path the causality flows.

From the directness perspective, a general concern is that temporal distance allows for additional, unconsidered (e.g., unmodeled) effects to occur, and so the *TE* is emphasized. Regardless of the complexity of a model, a model is always just a model and by definition it does not capture all aspects of the variable relationships (Edwards, 2013). In reality there are always intervening events such that with increasing time between measurements the chances are higher that unknown events are the proper causes of the dependent variable, rather than the mediator(s). Though a full discussion is too complex to engage in here, a similar view has been taken by philosophers such as, Woodwarth (2003). The inclusion of a mediator necessarily increases the minimum distance between *X* and *Y*, and the associated paths are necessarily correlational and require additional model assumptions, and if these assumptions do not hold then the estimates of the *IE* and *DE* are biased (Sobel, 2008). Additionally, one can manipulate *X* but not *M* at the same time without likely interfering with the proposed mediation process and thus potentially destroying it, and so the link between *M* and *Y* remains a correlational one.

Network models are an interesting example of an indirectness perspective on causation, and one that is taken to a relative extreme. In such models, a large number of variables cause one another, and possibly mutually so, e.g., insomnia may result in concentration difficulties and then work problems, which may then aggravate the insomnia due to excess worry, before ultimately resulting in a depressed state (Borsboom and Cramer, 2013). Another example of an indirectness perspective can be found in relation to climate change: Lakoff (2012) posted an interesting discussion and introduced the term “systemic causation” for causation in a network with chains of indirect causation. Many mediation models one can find in the psychological literature would qualify for the label of systemic causation, both in terms of the model (e.g., multiple connected mediators) and in terms of the underlying processes (e.g., changes in neurotransmitters underlying changes in behavior). Somewhat akin to the effect vs. model testing perspectives, if the additional statistical and theoretical assumptions hold then the benefit is a fuller and more precise picture of the variable relationships, but if they do not then statistical analyses will yield biased estimates and the inferences drawn made suspect.

The two perspectives make sense for the example application from the simulation study. From the directness perspective, as the number of time points increases it becomes increasingly difficult to claim that *X* has a causal effect on *Y*. It is easy to make such claims for *T* = 3, but for a large number of time points such as, *T* = 50 or 100, claims of causation are most relevant to the mediators most proximal to *Y* (alternatively, to those shortly following *X*). In contrast, for the indirectness perspective, a systems interpretation of causality makes perfect sense for time series. The autoregressive process does have causal relevance, and the identification of such a long chain of effects would likely be considered compelling evidence of causation.

Thus, indirectness and distance make a causal interpretation stronger from one perspective, whereas they make a causal interpretation less convincing from another perspective. These two perspectives are not in direct contradiction—they simply focus on different aspects of the same reality and reflect different needs and concerns. In the case of directness, the criterion is a minimizing ambiguity about whether or not there is an effect of *X* on *Y*. In contrast, in the case of adopting an indirectness perspective, the primary criterion is maximizing information about the process and thus about intermediate steps because it makes the causal process more understandable.

## Hypothesized vs. alternative explanations

Our final pair of perspectives refers to whether one is primarily interested in a confirmatory test of a mediation hypothesis about the relationship between two variables or whether one would rather test one or more other explanations that would undermine a mediation claim. Loosely, the difference between these two perspectives is that the former focuses on showing that a mediation explanation is appropriate, and the latter focuses on showing that alternative explanations are not.

In practice this distinction can be a subtle one, as it is always necessary to control for confounders, but there are considerable differences in the information acquired and required for these two perspectives, as well as the amount of effort invested and what is attended to Rouder et al. (2016).

For mediation, researchers generally work with a theory-derived mediation hypothesis and collect data that allows them to test the null hypothesis of no mediation. It is a search for a well-defined form of information, and further the search is considered complete when that information is obtained. If the null hypothesis of no relationship is rejected, the mediation claim is considered to be supported and the case closed. If it is not rejected, explanations are generated as to why the study failed, and the hypothesis is tested again (ideally in a separate study, but this also manifests as including unplanned covariates in the statistical models). Alternative explanations are often not generated or tested if the null hypothesis of mediation is rejected. This is an intriguing asymmetry between the two possible outcomes of a study—supportive results are accepted, unsupportive results are retested.

A somewhat different approach is to formulate alternative explanations for a significant effect that are in conflict with a mediation claim. The simplest and most common means of doing this is to include additional covariates in Models 2 and 3 that are competing explanations for the relationships between the three variables, or to experimentally manipulate these explanations as well. In cases where temporal precedence is not clear such as, in observational data or when there are only two time points, it is also useful to consider alternative variable orders, e.g., treating *X* as *M* or *M* as *Y*. Another approach is to assume that there are unmeasured confounders that bias the estimates and necessitate examining parameter sensitivity (VanderWeele, 2010). Still another is to test the proposed mediator as a moderator instead (a distinction which is itself often unclear; Kraemer et al., 2008) or as a hierarchical effect (Preacher et al., 2010).

Referring to the time series example, it was simply a test of an autoregressive model with a single lag and the power to detect such small effects in a constrained serial mediation model, but in practice it would also make sense to consider a moving-average model, where the value of an observation depends on the mean of the variable and on a coefficient associated with the error term (Brockwell and Davis, 2013). Loosely, the residuals might “cause” the values of subsequent time points, and are not simply measurement errors but new and unrelated inputs specific for the time point in question.

As with each previous pair of perspectives, both perspectives have advantages and disadvantages. Focusing on confirmation has the general advantages of simplicity and expediency by utilizing past research to direct future research, with a relatively clearly defined set of criteria for what counts as supporting evidence. There are also cases where it is not necessary to exhaust all alternatives, and instead simplicity and sufficiency of an explanation are valued more strongly. However, this perspective comes with the risk of increased false-positives and a narrow search for explanations for relationships between variables because what is considered is determined in part by what is easy to consider. Finding that one explanation works does not prove there are no other—and possibly better—explanations, and a model is always just a model (Edwards, 2013).

Focusing on competing hypotheses has the advantage of potentially providing stronger evidence for a mediation claim by way of providing evidence that competing hypotheses are not appropriate. Conversely, when a competing hypothesis cannot be ruled out easily, it may turn out to be a better explanation than a mediation model upon further research. However, there are a few very strong limitations regarding competing evidence. The first is that for every explanation, there are an infinite number of competing explanations that are all equally capable of describing a covariance matrix. Some are ignorable due to their sheer absurdity, but there are still an infinite number of reasonable alternative explanations (for example, it is easy to generate a very long list of explanations for why self-esteem and happiness correlate) and criteria for evaluating these explanations are often unclear or extremely difficult to satisfy. Further, it is often impossible to estimate alternative statistical models because of the limited information provided by only a small set of variables (e.g., factors are difficult to estimate with a small number of indicators). Similarly, estimating a very large number of complicated interacting variable relationships may require sample sizes that are not realistic.

## A note regarding philosophical considerations

Before turning to our discussion, we wish to note that philosophical views on causality differ with respect to whether a total effect is implied or necessary, and that there is substantial overlap between the philosophical views and our discussion of directness vs. indirectness distinction. We rely on a chapter by Psillos (2009) in the *Oxford Handbook of Causality* for a brief discussion of philosophical views, but see White (1990) for an introduction for psychologists.

In Humean regularity theories, *X* is a cause if it is regularly followed by *Y*. This suggests a total effect as a condition for *X* being a cause of *Y*. In a deductive-nomological view attributed to Hempel and Oppenheim, for *X* to be a cause it needs to be connected to *Y* through one or more laws so that *X* is sufficient for *Y*. Sufficiency would again imply a total effect, albeit possibly a very small one, because there may be multiple sufficient conditions. Only when a condition is at the same time sufficient and necessary can one expect a clear relationship.

Another view is formulated in the complex regularity view of Mackie (1974) and his INUS conditions. According to this view a cause is an *I*nsufficient but *N*on-redundant part of a condition which is itself *U*nnecessary but *S*ufficient for the effect. In other words, a cause is a term (e.g., *A*) in a conjunctive bundle (e.g., *A and B and C*), and there can be many such conjunctive bundles that are each sufficient for the effect. This expression is called the disjunctive normal form (e.g., *Y* if and only if *A and B and C* or *D and E* or *F and G* or *H* or *I*). This form does not imply a total effect of *X* on *Y* (e.g., *A* as *X*), because the disjunctive normal form may be highly complex and may therefore not lead to *X* and *Y* being correlated, while *X* is still accepted as a cause because it is part of that form. In other words, the relationship between a cause and the event to be explained is such that a cause can occur either with or without the event and vice versa. The INUS view is consistent with indirectness and systemic causation, whereas Humean regularity theory is better in agreement with directness of causes.

## Discussion

From the above discussion of the various perspectives we wish to conclude that there is not just one way to look at mediation. Researchers may approach mediation with or without an *a priori* hypothesis, or may focus on either a global model or a specific effect that derives either from the global model or that is estimated from separate regression analyses. A researcher may value directness or indirectness as causal evidence, or may prefer effect-focused or significance-focused tests. Researchers may further focus on hypothesized or competing alternative explanations when testing for mediation. Each pair of perspectives has associated advantages and disadvantages, and which is to be preferred depends on the nature of a given study or topic of interest.

The perspectives we have discussed here do not exhaust all common perspectives. Another common pair is a practical vs. a theoretical goal for testing a mediation claim. The aim of a mediation study can either be to find ways to change the level of the dependent variable, or the aim can be to understand the process through which the independent variable affects the dependent variable, or the purpose of the research may be prediction. Mediation can help to understand a process and advance a theoretical goal even when the total effect is negligible, but from a practical point of view, mediation is not helpful for such a case unless there is an easily addressed suppression effect or *Y* represents an important outcome such as, death. For applied settings where affecting change by way of an intervention of some sort, a direct effect or an unsuppressed large indirect effect is in general much more useful.

Another example is that the concept of mediation remains somewhat ambiguous despite the clarification provided by Baron and Kenny (1986). That mediation explains the relationship between *X* and *Y* can mean two things: (1) Mediation *explains values of Y* as indirectly caused by values of *X*. (2) Mediation *causes the relationship between X and Y*. Following the second interpretation, the relationship itself (or absence of relationship) is explained by values of *M*. Here, we have interpreted the concept of mediation in the first sense. Note that the second way of understanding mediation is also commonly considered to be *moderation*, where *M* is supposed to explain why there sometimes is a relationship between *X* and *Y* and sometimes there is not (or why the strength of the relationship varies). The MacArthur approach provides some clarification regarding the latter sense (the approach is named after a foundation; Kraemer et al., 2002, 2008), and notably it adds an interaction term between *X* and *M* to Model 3. The approach specifies that if *X* precedes *M*, there is an association between *X* and *M*, and there is either an interaction between *X* and *M* or a main effect of *M* on *Y* then *M* is said to mediate *Y*. In contrast, if there is an interaction between *X* and *M*, but no main effect of *M* on *Y*, then *X* is said to moderate *M*. In short, the approach specifies that a statistical interaction can still reflect mediation (see also Muller et al., 2005; Preacher et al., 2007). The approach further focuses on effect sizes over NHST, and states that causal inferences should not be drawn from observational data for reasons similar to those we provide in the discussion of the hypothesized vs. alternative explanations section. The approach also explicitly treats the indirect effect as only potentially causal, arguing that the Baron and Kenny approach to mediation and moderation can potentially bias the search for explanations because of its assumption that the causal process is already known but must only be tested. The MacArthur approach then seems to favor (or is at least mindful of) some of the specific perspectives we have discussed here, and it remains to be seen what the impact is of the approach on mediation and moderation practice and theory.

We have discussed mediation at a rather abstract, general level, and some of the details of the different perspectives we have discussed here are not always relevant to specific statistical analyses. In keeping with common practices we have utilized parametric mean and covariance-based approaches for our discussion, but median-based approaches to mediation have been proposed (e.g., Yuan and MacKinnon, 2014), and for such approaches the notion of global model testing by way of comparing the fit of different SEMs is largely irrelevant in a frequentist framework (though it may be done within a Bayesian framework; Wang et al., 2016). For network analysis, the strong focus on indirectness of effects within a larger system with a very large number of variables that each may be treated as *X, M*, or *Y*, renders the issue of a specific mediation hypothesis or a total effect irrelevant.

On the other hand, while we have discussed each perspective as independent views, there are obvious intersections between them and ample reasons to adopt the opposing perspective in some cases, or even both for the same study. For example, when working with a global model, specific effects within the model vary in how trustworthy they may be considered. Those effects that are considered less trustworthy can be interpreted more from a directness perspective because of the ambiguity regarding their effects, and those that are uncontroversial can be interpreted from an indirectness perspective. Confidence intervals and NHST also make use of the same information and if interpreted dichotomously (reject vs. not reject) the results will not differ. There are also intersections across pairs as well, e.g., testing competing explanations is facilitated by adopting a global model-focused approach, and the issue competing explanations in general provides much of the rationale for preferring a directness perspective on causation.

We wish to include a cautionary note concerning causality before concluding. A mediation hypothesis is a causal hypothesis (James and Brett, 1984), but we realize that a causal relationship is difficult if not impossible to prove in general, let alone in the complex world of the social sciences (Brady, 2008). Further, the statistical models used to test mediation are not inherently causal—they are simply predictive or descriptive, and the *b* path is necessarily correlational (Sobel, 2008). That the data are in line with the hypothesis and even that several alternative explanations can be eliminated does not prove causality. It does not follow from the combination of the two premises “If A then B” (if *M* mediates then the null hypothesis of no indirect effect is rejected) and “B is the case” (null hypothesis rejected) that “A is the case.” (M mediates; i.e., the fallacy known as affirming the consequent). Instead, modus tollens (i.e., “B is not the case”) is a valid argument for the absence of A, so that one may want to believe that A is ruled out in the absence of B. Although the reasoning is logically correct, the problem with mediation analysis is that “B is not the case” in practice is simply a probabilistic non-rejection of a null hypothesis and does not directly implicate the truth of any other claim.

## Conclusion

Human behavior and psychology emerges from dynamic and complicated systemic effects that are impossible to capture completely, and researchers choose what must be understood for a given problem—what fraction of the network of interacting variables is most relevant—and so which perspective to adopt. Ultimately, mediation analysis is simply a tool used for describing, discovering, and testing possible causal relationships. How the tool is used (or not used) and what information is most relevant depends on the problem to be solved and the question to be answered.

## Author contributions

RA was responsible for most of the writing, in particular any revisions and the introduction and discussion. PD provided most of the core points involved in the discussion of each perspective.

### Conflict of interest statement

The authors declare that the research was conducted in the absence of any commercial or financial relationships that could be construed as a potential conflict of interest.
